# Social calls of flying big brown bats (*Eptesicus fuscus*)

**DOI:** 10.3389/fphys.2013.00214

**Published:** 2013-08-16

**Authors:** Genevieve S. Wright, Chen Chiu, Wei Xian, Gerald S. Wilkinson, Cynthia F. Moss

**Affiliations:** ^1^Department of Biology, University of MarylandCollege Park, MD, USA; ^2^Department of Psychology, University of MarylandCollege Park, MD, USA; ^3^Institute for Systems Research, University of MarylandCollege Park, MD, USA

**Keywords:** big brown bat, communication, competition, *Eptesicus fuscus*, foraging, inter-bat distance, social calls

## Abstract

Vocalizations serving a variety of social functions have been reported in many bat species (Order Chiroptera). While echolocation by big brown bats *(Eptesicus fuscus)* has been the subject of extensive study, calls used by this species for communication have received comparatively little research attention. Here, we report on a rich repertoire of vocalizations produced by big brown bats in a large flight room equipped with synchronized high speed stereo video and audio recording equipment. Bats were studied individually and in pairs, while sex, age, and experience with a novel foraging task were varied. We used discriminant function analysis (DFA) to classify six different vocalizations that were recorded when two bats were present. Contingency table analyses revealed a higher prevalence of social calls when males were present, and some call types varied in frequency of emission based on trial type or bat age. Bats flew closer together around the time some social calls were emitted, indicating that communicative calls may be selectively produced when conspecifics fly near one another. These findings are the first reports of social calls from flying big brown bats and provide insight into the function of communicative vocalizations emitted by this species.

## Introduction

Since the pioneering studies of Griffin and Webster, it has been recognized that many bats produce high frequency calls and use information carried by returning echoes to localize objects in their environment (Griffin, 1958; Griffin et al., 1960). Research has also shown that bats emit vocalizations in social contexts (see Fenton, 1985; Pfalzer and Kusch, 2003). For example, Suthers (1965) described a distinctive call produced by fishing bats (*Noctilio leporinus*) to avoid in-flight collisions. In addition, vocalizations produced by bats have been reported to serve mating-related functions (e.g., Bradbury, 1977: *Hypsignathus monstrosus*; Lundberg and Gerell, 1986: *Pipistrellus pipistrellus*; Davidson and Wilkinson, 2004: *Saccopteryx bilineata*), to recruit conspecifics (e.g., Wilkinson and Boughman, 1998: *Phyllostomus hastatus*; Arnold and Wilkinson, 2011: *Antrozous pallidus*), to respond to bats calling from a roost (e.g., Chaverri et al., 2010: *Thyroptera tricolor),* to avoid physical aggression (Leippert, 1994: *Megaderma lyra*), and to defend foraging patches (e.g., Rydell, 1986: *Eptesicus nilssoni*; Barlow and Jones, 1997: *Pipistrellus pipistrellus*). Despite these studies, few examples of communicative vocalizations emitted by flying, foraging bats have been reported. Examining such vocalizations, in concert with information about bat sex, age, foraging context, and inter-bat interactions, can provide insight into the functions of social calls in bats.

Social calls emitted by bats during flight might serve to repel or attract other foragers. For example, calls produced by *Pipistrellus pipstrellus* when food density is low have been shown to repel conspecifics (Barlow and Jones, 1997), whereas calls emitted by female *Phyllostomus hastatus* coordinate group foraging (Wilkinson and Boughman, 1998). Alternatively, calls might influence mating and therefore should occur most frequently at the time of year when animals are engaged in reproductive behaviors. For example, male *Tadarida brasiliensis* produce songs during a limited period each spring (Bohn et al., 2009). Finally, calls with an appeasement function (Gadziola et al., 2012) would be expected to be produced by vulnerable individuals, such as juveniles, to avoid aggressive encounters with other bats, as has been proposed for calls emitted by *Megaderma lyra* (Bastian and Schmidt, 2008).

*Eptesicus fuscus* is a temperate, aerial-hawking insectivore that is widespread in North America (Kurta and Baker, 1990). Female *E. fuscus* form maternity colonies in the spring and early summer, and the bats “swarm” (Fenton, 1969) and mate at hibernation sites before hibernating for the winter. This species forms non-random associations with roost-mates (Willis and Brigham, 2004; Metheny et al., 2008), and multiple individuals can be found foraging at the same site, indicating that bats have opportunities to communicate while foraging. Two studies have reported that *E. fuscus* can learn a novel foraging task or food location by interacting with knowledgeable conspecifics (Gaudet and Fenton, 1984; Wright et al., 2011).

Echolocation by *E. fuscus* has been studied extensively (e.g., Simmons and Vernon, 1971; Masters et al., 1991; Surlykke and Moss, 2000). Some research indicates that echolocation signals themselves can serve a communicative function, such as revealing information about identity, age, and sex (Masters et al., 1995; Kazial and Masters, 2004; Grilliot et al., 2009; Jones and Siemers, 2010; Knörnschild et al., 2012). However, most studies of social calls in this species have focused on mother-infant communication or vocal development (e.g., Gould, 1971, 1975; Gould et al., 1973; Moss, 1988; Monroy et al., 2011). A recent study of roosting or crawling bats indicated that social call production varies with behavioral context (Gadziola et al., 2012), but, to date, we know of no description of social calls from flying big brown bats, although Barbour and Davis (1969) noted that *E. fuscus* are known to emit an “audible chatter” (p. 130) when flying near each other.

In this study, we document the occurrence and the context of social calls emitted by big brown bats flying together in a large behavioral test room. We manipulated context by varying prey-capture skill level, age, and sex of bat pairs and then used recordings of high-speed video and audio to determine the position of each individual before and after emitting social calls. If calls served a mating related function, we expected them to be emitted primarily in late August or September when spermatogenesis peaks and mating in this species typically begins (Kurta and Baker, 1990) and to be produced by males flying in the presence of females. If calls served to recruit or repel individuals to or from a food source, we expected a higher rate of calls when at least one skilled forager was present. Finally, we predicted that calls related to appeasement would be most common when juveniles were present. Here we test these predictions and describe the repertoire of social calls emitted by flying big brown bats.

## Materials and methods

### Subjects and experimental set-up

Thirty-six *Eptesicus fuscus* obtained from the wild under a Maryland Department of Natural Resources collecting permit and two born in captivity served as subjects in this study. This research was conducted with approval from the Institutional Animal Care and Use Committee at the University of Maryland. At the time of testing, 24 animals were adults (≥1 year old; 17 F, 7 M), and 14 were juveniles (estimated ages at start of testing: 21–51 days (*X* ± *SD* = 34 ± 10); 6 F, 8 M). Based on their ages, the juvenile bats should not have been reproductively capable during most or all of the experimental period. Bats always had access to water and were maintained on a reverse 12:12 h light:dark cycle (lights off from 08:30 to 20:30). When not flying, they were housed in cages containing three to four bats each.

We flew pairs of big brown bats in the presence of a single, non-shareable prey item (tethered mealworm—larval *Tenebrio molitor*) in a 7 × 6 × 2.5 m anechoic flight room. As bats flew, we recorded 8 s segments of synchronized audio and video data using two high-speed (240 frames/s in 2005–2006; 250 frames/s in 2007) infrared-sensitive video cameras (in 2005–2006: Kodak MotionCorder Analyzers, Model 1000, Eastman Kodak Company, San Diego, CA, USA; in 2007: Photron PCI-R2, Photron USA, Inc., San Diego) and two ultrasound-sensitive microphones (UltraSound Advice, London, UK) amplified (UltraSound Advice) and recorded at 250 kHz/channel (Wavebook, IOTech, Cleveland, OH, USA). The room was lit with low-intensity and long wavelength overhead lighting (>650 nm, red filters, Reed Plastics, Rockville, MD, USA) and two red light-emitting diode (LED) headlamps to minimize availability of visual cues [see Chiu et al. (2008) and Wright et al. (2011) for additional details]. Recordings from 415 one-bat and 528 two-bat trials involving 83 pairs of bats were then examined.

Bat pairs fell into three categories: (1) one individual had learned to take the tethered mealworm, while one was naïve (mixed trial type; July–September 2006 and July–August 2007; 36 pairs), (2) both individuals were naïve (naïve trial type; July–September 2006 and July–August 2007; 40 pairs), or (3) both individuals had learned to take tethered mealworms (skilled trial type; July–August 2005 and July–August 2006; 7 bat pairs). While some naïve individuals in mixed trials began to learn the task, previously naïve individuals were no longer paired with other bats once they learned to capture the mealworm (Wright et al., 2011). We recorded all individuals in paired bat trials, and each bat flew with an average of 4.5 other bats (range: 1–11 partners; median: 4 partners). A test day began with both bats being released simultaneously (skilled pairs) or in some cases with a naïve bat resting on the wall when another bat was released (naïve and mixed pairs). For skilled and mixed pairs, we recorded prey capture and the previous 8 s. On a given test day, once the mealworm was taken, another was immediately presented to the same pair of bats until 10–20 mealworms had been consumed. For naïve pairs, bats were flown for a fixed period of time (7 min.) based on the time it took trained bats to consume 10–20 mealworms, and 8 s recording segments were saved throughout this time period, as described in Wright et al. (2011). Skilled pairs were captured in between each mealworm presentation, while mixed and naïve pairs flew freely during this time. Bats occasionally landed on the flight room wall during trials but were usually flying. In addition to two-bat trials, we recorded single-bat trials from 22 naïve and eight skilled bats. Please see Chiu et al. (2008) and Wright et al. (2011) for additional details.

### Identification and classification of call types

By inspecting spectrograms and listening to audio files slowed by a factor of 10–20, we identified calls that differed in time-frequency structure from frequency-modulated (FM) echolocation calls produced by big brown bats. We did not employ a frequency cut-off regarding which calls to include, but we excluded vocalizations resembling buzzes [feeding buzz pulses drop below 20 kHz, have short duration (<1 ms), and have short pulse interval (PI; <8 ms)] because these calls were typically produced when bats were feeding, landing or investigating objects in the room, and their potential social function could not be separated from echolocation function. Other, low frequency calls were, however, included in the data set presented here. We considered emission of calls only in the presence of conspecifics as evidence that calls serve a social function.

**Figure 1:**
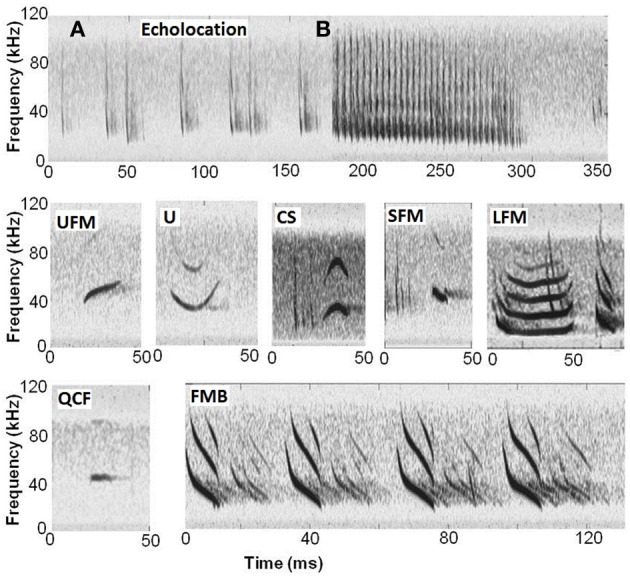
**Calls recorded in a flight room. (A)** Standard echolocation calls (two bats flying); **(B)** feeding buzz with the second bat echolocating; UFM, upward frequency-modulated; U, U-shaped; CS, chevron-shaped; SFM, short frequency-modulated; LFM, long frequency-modulated (double-LFM showing long and short varieties of the call type); QCF, quasi-constant frequency; and FMB, frequency-modulated bout with only the initial FM sweeps shown (four pulses and their echoes are shown). Note that for several of the examples above (e.g., CS, SFM, and LFM), echolocation calls from the other bat present in the trial are also visible.

We first categorized calls by consistent patterns in time-frequency structure. This method resulted in seven call types: (1) upward frequency-modulated (UFM)—end frequency exceeds start frequency by ≥5 kHz without additional change in frequency; (2) U-shaped (U)—dominant frequency decreases by ≥5 kHz, then increases again to between 50 and 150% of the start frequency; (3) chevron-shaped (CS)—dominant frequency increases by ≥5 kHz, then decreases again to between 50 and 150% of the start frequency; (4) short frequency-modulated (SFM)—short duration, narrow bandwidth calls with ending frequency ≥18 kHz, duration ≤6 ms, and bandwidth ≤20 kHz; (5) long frequency-modulated (LFM)—an initial downward sweep, and duration (3.75–82.7 ms) longer than typical echolocation calls produced by big brown bats in a confined space (>3.7 ms, mean duration of echolocation calls in our single bat recordings)—these calls appeared in two varieties: short (chirp-like FM sweeps virtually always paired with a long LFM) and long (elongated quasi-constant frequency portion after initial frequency drop) and often occurred in pairs or trios; (6) quasi-constant frequency (QCF)—dominant frequency is within 5 kHz of the start frequency; and (7) frequency-modulated bout (FMB)—a sequence of 3–4 frequency-modulated (FM) sweeps that were longer in duration than typical echolocation calls (mean duration of FMB pulses: 9.2 ms, compared with echolocation call durations ≤4 ms) sometimes followed by several short, buzz-like calls (short duration calls with relatively short PI; Figure 1). FMB refers to a specific pulse type and the fact that it occurs in a sequence of 3–4 such pulses. Not all FMBs were followed by buzz-like pulses; therefore, the presence of such pulses was not considered a defining characteristic of this call type.

To quantify the accuracy of this call classification system, we conducted a discriminant function analysis (DFA) assuming unequal covariances and using start frequency (kHz), end frequency (kHz), mid-frequency (frequency in the middle of the call's start and end time; kHz), and call duration (ms). For call types with more than one pulse (FMB, some LFM), we took the mean values of all pulses within the sequence and used these data in the DFA. We did not include the short, buzz-like calls that often occurred at the end of FMB, for the reason noted above. Due to the small number of U calls recorded (*n* = 26), we excluded this call type from the DFA and all subsequent quantitative analyses.

### Caller identification and call context

To rule out the possibility that calls of a given type were produced exclusively by one individual, we calculated the minimum number of individuals emitting each call type by examining the number and composition of pairs from which calls were recorded. In addition, we used a combination of video and audio data to identify, when possible, which bat had emitted each vocalization using the following criteria: (1) the social call was visible in the spectrogram of both audio channels, (2) at least one bat was in view of both cameras during the time the call was emitted, and (3) both individuals were identifiable during the trial (see Chiu et al., 2008). Particularly in naive bat trials wherein no bat was catching the prey, we often did know which bat was which during a given recording: we might determine that one social call was emitted by “Bat A” while another call was emitted by “Bat B,” but we could not always determine whether Bat A was the adult female or the juvenile male (for example) in that recording. Therefore, caller identification was not possible for all calls. For call types emitted by more than five known callers, we compared the number of callers of each sex with the proportion of bats we tested that were female (61%) or male.

To determine the context in which calls were given, we investigated whether call occurrence was independent of trial type, bat age, and bat sex. Because we could not always determine which bat emitted a call, and we recorded few trials per pair in some cases (range: 1–25 trials per pair; median: 5 trials), we accounted for variation in the number of calls emitted by each individual by examining the data on a per-trial basis. Specifically, we compared the number of trials containing at least one instance of a given social call type. We excluded juvenile-juvenile trials from these analyses because all 25 trials included one bat in common and only one such trial contained any social call. Data included trials from every combination of sex (female–female: *N* = 126 trials; female–male: *N* = 256 trials; male–male: *N* = 121 trials) and trial type (naïve: *N* = 181 trials; mixed: *N* = 170 trials; skilled: *N* = 152 trials).

We examined the relationship between each factor (age, sex, and foraging experience) and call prevalence, using contingency tests, for each call type. For SFM, we found a significant interaction between trial type and sex, so we tested for effects of trial type within trials with the same sex combination. Because all bats tested in skilled trials were adults, we could not test for age effects in those trials. Instead, we tested for age (adult–adult: *N* = 69 trials; adult–juvenile: *N* = 282 trials) effects within naïve and mixed trials combined for UFM, CS, SFM, LFM, and QCF calls. We recorded too few FMB from naïve and mixed trials to conduct this analysis. Because tests regarding these factors were all drawn from the same data set, we used a sequential Bonferroni correction to assign significance for each of the 19 comparisons made. For call types with significant differences based on trial type or sex, we conducted pairwise comparisons (e.g., female-male vs. male-male trials, or naïve vs. skilled trials). For these comparisons, we used a sequential Bonferroni correction within each factor for each call type (three comparisons for each combination).

### Flight behavior

Using a custom Matlab program that allowed us to mark and plot the three-dimensional flight trajectories of each bat (see Chiu et al., 2008), we determined in-flight inter-bat distances between animals. We calculated inter-bat distances for the 1 s surrounding the time of social calls (mean of the 500 ms before the start and after the end of each social call), as well as the mean inter-bat distance for the entire 8 s trial in which each social call was recorded. Only video frames with both bats flying in the calibrated volume of the two cameras were included in the analyses. Therefore, animal position data was not available for every social call or for every frame within each 8 s recording, and we sometimes had less than 1 s of video position data surrounding a social call.

We had unequal and sometimes sparse numbers of recordings from each pair of bats and could not always determine caller identity. Therefore, we examined data on a per-trial (recording) basis and only included call types with position data available for 10 or more calls. We averaged the mean inter-bat distances for all calls of a given type within a single recording, and then used paired *t*-tests to compare mean inter-bat distance at the time of calls vs. entire 8 s recordings for each call type.

## Results

### Call classification

In 187 two-bat trials, recorded from 32 bats comprising 53 pairs, we identified seven distinct social call types shown in Table 1. We recorded a total of 764 vocalizations or call groups, henceforth referred to as social calls, which were distinct from echolocation calls. Only call types with at least 60 examples were included in the DFA; hence U calls were excluded.

**Table 1:** **Call parameter values for each call type**.

**Call type**	**Start frequency *X* ± *SD* (kHz)**	**Mid-frequency *X* ± *SD* (kHz)**	**End frequency *X* ± *SD* (kHz)**	**Duration *X* ± *SD* (ms)**	**Percentage of 528 recordings in which call(s) occurred**	**Total calls recorded**
Upward frequency-modulated (UFM)	48.0 ± 7.8	53.4 ± 6.7	62.8 ± 9.6	15.0 ± 4.8	8.5	140
U-shaped (U)	50.8 ± 7.4	42.8 ± 8.2	51.1 ± 10.8	16.9 ± 6.6	3.03	26
Chevron-shaped (CS)	47.7 ± 9.0	55.4 ± 8.3	44.3 ± 10.3	16.6 ± 5.4	6.06	92
Short frequency-modulated (SFM)	39.0 ± 5.5	30.8 ± 4.4	25.6 ± 4.4	3.5 ± 1.2	9.7	91
Long frequency-modulated (LFM)^#^	42.6 ± 9.1	21.7 ± 5.8	18.1 ± 4.8	23.8 ± 13.6	7.6	163 (223 pulses)
Quasi-constant frequency (QCF)	44.1 ± 12.0	43.7 ± 13.3	41.9 ± 14.0	12.7 ± 5.2	5.5	66
Frequency-modulated bout (FMB)^#^^∧^	69.2 ± 10.9	33.4 ± 8.1	17.3 ± 4.7	9.2 ± 0.8	35.2	186 (645 pulses)

# *The mean of all pulses within a call/bout was used when calculating means and SDs*.

∧ *Values are for the first 3–4 calls per bout and do not include the shorter duration, buzz-like calls that often follow*.

**Figure 2:**
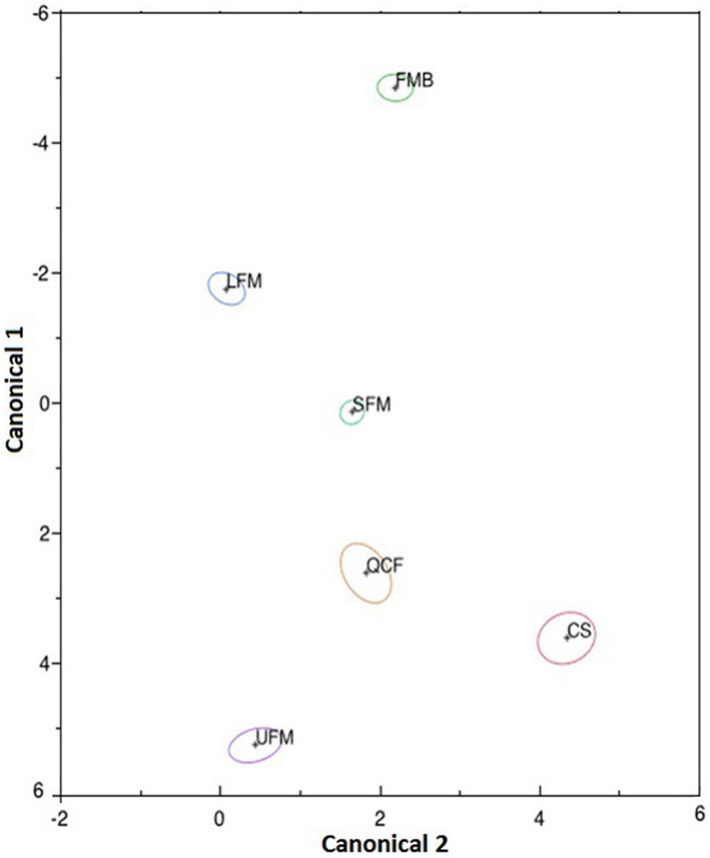
**Plot of the first two canonicals, which together explain 90.8% of the variation in the data, for each call.** Each point represents the centroid for a given call type. Ellipses show the 95% confidence interval around each centroid. See Table 2 for standardized coefficients that indicate how the call parameters contribute to canonical 1 and 2. Overall, 95% of calls were correctly classified to type. Call type abbreviations: UFM, upward frequency-modulated; CS, chevron-shaped; SFM, short frequency-modulated; LFM, long frequency-modulated; QCF, quasi-constant frequency; and FMB, frequency-modulated bout.

Considering that the results from cross-validation DFAs using half of the data for training were very similar (92–94% correct classification) to those using all of the data at once, we report the results from the entire data set. Based on the results of this DFA, 94.9% of calls were correctly classified [MANOVA: Wilk's lambda = 0.007, *F*_(20, 2419)_ = 413.03, *P* < 0.0001]. Individual call types were correctly classified as follows: UFM, 92.1%; CS, 93.5%; SFM, 96.7% LFM, 97.5%; QCF, 80.3%; and FMB, 99.5% (Figure 2). The first canonical dimension explained 80.6% of the variation, while the next three dimensions explained 10.2, 5.9, and 3.3%, respectively. Inspection of the standardized coefficients (Table 2), which indicate how the variables are weighted to form each canonical axis, indicates that most (91%) of the variation among call types is due to differences in frequency, given that duration contributes very little to the first two axes. Based upon the DFA results, we treated these call types as distinct for subsequent analyses.

**Table 2:** **Standardized coefficients for the discriminant function analysis**.

**Canonical**	**Start frequency**	**End frequency**	**Mid-frequency**	**Duration**
1	−1.386	0.819	0.803	0.129
2	−0.491	−1.411	2.095	−0.099
3	0.829	0.758	−0.732	−0.559
4	0.449	−0.007	0.177	0.837

The mean duration of FMB (not including buzz-like calls) was 79.8 ms, with an average of 3.47 calls per bout (virtually always 3 or 4 calls). The mean duration of LFM was 37.4 ms, with an average of 1.36 calls per sequence (110 single calls, 46 doublets, and seven triplets).

### Call context

Calls were produced at various times during 8 s recordings. Because recordings from skilled trials (and most mixed trials) ended with one bat taking the mealworm, the social calls recorded occurred during these 8 s segments. In naïve trials, no bat was taking the mealworm, so emitted calls were recorded at various 8 s intervals throughout the trial period.

**Figure 3:**
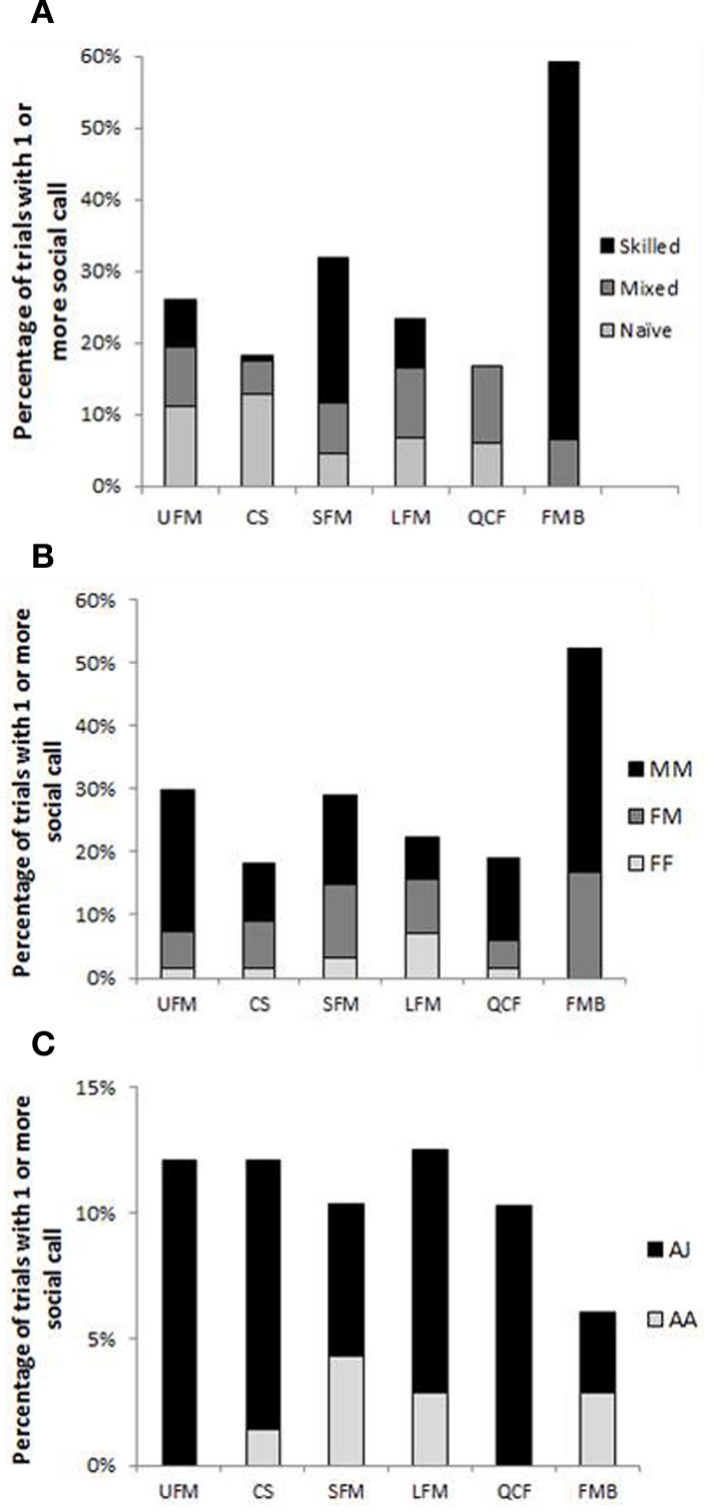
**Percentage of trials from each trial type (A), sex combination (B), and age combination (C) containing at least one instance of social calls of each type.** See Table 3 for related statistics. Because all skilled trials (*N* = 152) contained only adults, skilled trials are excluded from panel **(C)**. Mixed (*N* = 170) and naïve (*N* = 181) trials are mostly from adult–juvenile pairs, which is why calls from this age combination appear so much more common than social calls from adult–adult pairs in the figure. MM, male–male (*N* = 121); FM, female–male (*N* = 256); FF, female–female (*N* = 126); AJ, adult–juvenile (*N* = 282); and AA, adult–adult (*N* = 69 naïve and mixed trials) trial types. Call type abbreviations: UFM, upward frequency-modulated; CS, chevron-shaped; SFM, short frequency-modulated; LFM, long frequency-modulated; QCF, quasi-constant frequency; and FMB, frequency-modulated bout.

Contingency tests (Table 3) show that type of trial, sex, and age each influence when five of the six social call types (separated by the DFA) are produced. In general, more social calls were produced when males were present, with the highest prevalence of calls occurring in male-male trials. FMB were produced exclusively by males and were never recorded from a naïve pair of bats. With regard to trial type, CS calls were more common in naïve than mixed or skilled trials and more common in mixed than skilled trials, and QCF calls were more common in mixed and naïve trials than skilled trials. In addition, SFM and FMB were significantly more prevalent in skilled trials compared with naïve or mixed trials, and FMB were more common in mixed than naïve trials. With regard to sex, UFM, QCF, and FMB were significantly more common in male-male than female-male or female-female trials, and FMB were also significantly more common in female-male pairs vs. female-female pairs (no FMB was recorded from any female-female pair). Finally, UFM calls were more likely to occur in adult–juvenile vs. adult–adult trials (Table 3, Figure 3). LFM calls were emitted independent of trial type, sex, or age.

**Table 3:** **Differences in call prevalence based on trial type, sex combination, and age combination as determined by Pearson's Chi-Square statistics**.

	**UFM**	**CS**	**SFM^~^**	**LFM**	**QCF**	**FMB**
Trial type	—	**N > Mi > S**	**S > Mi S > N**	—	**Mi > S N > S**	**S > Mi > N**
Sex	**MM > FM MM > FF**	—	—	—	**MM > FM MM > FF**	**MM > FM > FF**
Age^∧^	**AJ > AA**	—	—	—	—	N/A

*Bold lettering indicates comparisons that are significant after a sequential Bonferroni correction. > indicates that the given call type was more common in the context to the left of the symbol.—indicates no significant difference for that comparison. N, naïve; Mi, mixed; and S, skilled trial type; MM, male–male; and FM, female–male; and FF, female–female trials; AA, adult–adult; and AJ, adult–juvenile. See Figure 3 for distribution of calls across trial types and sex and age combinations*.

∧ *Data pertaining to age refers only to naïve and mixed trial types*.

~ *Because we found a significant interaction between sex and trial type for SFM, we tested for type effects within female–male (FM) and male–male (MM) trials separately. The data shown above for SFM refer to FM trials; there was no significant difference in trial type within MM trials only, and we had insufficient data to test within FF trials*.

Based on position data, we assigned 335 calls of the six types separated using the DFA to a specific vocalizing bat. Social calls were emitted by males and females, and juveniles and adults. These 335 calls were attributed to 14 individuals (six juveniles initially naïve to foraging task and eight skilled adults; nine males and five females). Of these calls, UFM were emitted by six males (three juveniles, three adults) and no female; CS were emitted by four males and one female (four juveniles, one adult); SFM were produced by four males and three females (all adults); LFM were given by two males and three females (two juveniles, three adults); QCF were emitted by two males (one juvenile, one adult) and no female; and FMB were emitted by six males (one juvenile, five adults) and no female. Males were significantly more likely to emit UFM (*N* = 32 calls) and FMB (*N* = 168 calls) calls (*X*^2^_1_ = 9.4, *P* = 0.002 for each). Each call type was emitted by at least six individuals (based upon calls attributed to a certain bat and on bat pair composition), and with the exception of SFM, which were never assigned to a juvenile, every call type was emitted at least once by a juvenile, an adult, and a male.

### Flight behavior response to calls

**Figure 4:**
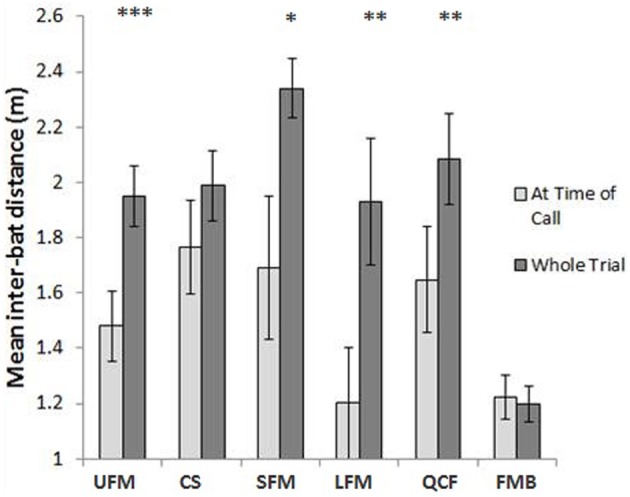
**Mean inter-bat distances before and after (“at time of call”) social calls were emitted and for trials containing these types of social calls overall.** ^*^ Indicates *P* < 0.05, ^**^ indicates *P* < 0.01, and ^***^ indicates *P* < 0.0001. Error bars represent one standard error. Call type abbreviations as in Figure 3. The mean inter-bat distance for entire trials is smaller for trials containing FMB compared with other call types because almost all FMB were recorded from skilled trials, and skilled bats competing for prey tend to fly closer together and exhibit increased following/chasing behavior compared with naïve bats (Wright et al., 2011). The closer distances are not necessarily related to FMB production.

Bats flew closer together around the time some call types were produced. Analyses show that UFM, SFM, LFM, and QCF were produced when individuals flew near each other. Bats flew significantly closer during the 1 s surrounding emission of these calls compared with complete recordings for UFM [*N* = 61 calls, paired *t*_(28)_ = 4.85, *P* < 0.0001], SFM [*N* = 55 calls, paired *t*_(26)_ = 2.34, *P* = 0.028], LFM [*N* = 25 calls, paired *t*_(7)_ = 4.40, *P* = 0.0031], and QCF [*N* = 25 calls, paired *t*_(15)_ = 2.97, *P* = 0.0096; Figure 4]. When most LFMs were produced, at least one bat was resting on the wall or out of camera view. Both bats were flying and in view of the cameras when only 15% of LFMs were emitted, so the data pertaining to inter-bat distance for this call type represents only a small portion of LFMs recorded in this study. We found no significant difference regarding inter-bat distance for CS [*N* = 41 calls, paired *t*_(19)_ = 1.68, *P* = 0.11] or FMB [*N* = 72 calls, paired *t*_(45)_ = 0.347, *P* = 0.73; Figure 4].

## Discussion

Vocal interactions mediate a variety of behaviors in bats (see Fenton, 1985), yet there have been relatively few descriptions of social calls emitted by flying bats, and even fewer where the identities and flight paths of individuals were known. In this paper we quantitatively differentiate six types of social calls from pairs of flying big brown bats, *Eptesicus fuscus*, and find that they occur nonrandomly depending on several factors. Each call type was emitted by several individuals, and prevalence of some call types differed depending on trial type, sex, and/or age. Some call types were also emitted more often when bats were in close proximity or when bats skilled at prey capture were flying, indicating that some calls likely influence foraging behavior as described below.

### Call context and flight behavior

For call types that covaried with sex (UFM, QCF, and FMB), trials with more than one male were always more likely than female-only trials to contain social calls, with male-male trials yielding the highest prevalence of social calls. Frequency-modulated bouts (FMB) were produced exclusively by male bats. Despite this male bias in call production, we did not find evidence to support an exclusive mating-related function for any call type. First, we found no call type in September that was not also recorded in July and August. In Maryland, the peak of spermatogenic activity for *E. fuscus* is in August, and mating occurs between September and March (Kurta and Baker, 1990). While it is possible that captive bats might not maintain mating seasonality, our captive bats show a marked decrease in activity during the time they would naturally hibernate, indicating that they are still influenced by seasonal changes. Second, calls were emitted with either sex present rather than only in the presence of the opposite sex.

We did find support for the hypothesis that some calls are related to foraging. Specifically, SFM and FMB were emitted more frequently in trials in which bats had experience taking tethered insects. Considering that only one prey item was available, bats were actively competing for food, making it unlikely that these calls served to recruit conspecifics, as has been reported for *Phyllostomus hastatus* (Wilkinson and Boughman, 1998). Notably, we recorded FMB exclusively when at least one bat was knowledgeable in the foraging task. While additional work is needed to reveal the role of FMB, this call may serve a food defense function, as was demonstrated for a foraging-related social call produced by pipistrelle bats (Barlow and Jones, 1997).

Bats flew closer together 500 ms before and after the production of UFM, SFM, LFM, and QCF than during the 8 s recordings containing these calls (Figure 4). The tendency of bats in this study to fly closer together when emitting social vocalizations may indicate that they selectively produce calls when they are near a conspecific, or that there is a greater need for communication when flying in close proximity. For instance, if a call's function is food-related, call emission might not be necessary unless the competitor is close to the caller or the prey item. If the function of a call is to warn another bat to keep its distance or to reduce potential aggression, the same idea would hold true.

While some call types appear to be foraging-related, CS calls were recorded significantly more often in trials with two naïve bats, and bats did not fly closer together before and after emission of CS calls compared with other times. Higher prevalence of this call type in naïve trials (when no prey capture occurred) indicates that its occurrence is not positively related to foraging. Instead, foraging situations may reduce the frequency of its emission, possibly because bats are instead producing other foraging-related social calls. Additional possible functions of CS calls include appeasement or conveying aggression (e.g., Leippert, 1994; Gadziola et al., 2012), but further research is needed to determine their purpose.

### Age and call prevalence

While the data relating inter-bat distance to call type can include only events when both bats were flying and in view of both cameras, many calls were emitted when at least one bat was out of view (either flying or resting on the wall). Anecdotally, we observed juvenile bats resting on the wall emitting social calls, often audible to the human ear, each time the other bat approached it as it circled the room. Given that LFM was the only call type with a mean end frequency below 20 kHz (Table 1) that was commonly recorded when juveniles were present, it is likely that many of these calls were LFMs, which closely resemble calls recorded by Gadziola et al. (2012) in an appeasement context. Gadziola et al. (2012) state that appeasement calls “appear to promote social contact” between individuals (p. 11). When we recorded LFMs, both bats were flying and visible during call emission for only a small percentage of calls. Considering our observations and the results in Gadziola et al. (2012), it is possible that juveniles resting on the wall were emitting appeasement calls when approached by flying adults. It should be noted, however, that regardless of the function of LFM calls, they are not emitted exclusively by juveniles, and there was no significant difference in LFM prevalence in adult–juvenile compared to adult–adult trials. While the structure of LFM calls resembles that of isolation calls produced by *E. fuscus* pups, our findings do not indicate that this call is age-limited. Emission of isolation calls in *E. fuscus* is reported to decline by week 4 (Moss, 1988; Monroy et al., 2011), yet 49% of the 45 LFM calls positively attributed to an individual bat were produced by adults, and 85% of trials (*n* = 40) containing LFM calls were recorded from bats >28 days of age, including 30% of trials with only adult bats present.

QCF calls were never recorded in adult-only trials, while all call types were recorded in adult–juvenile trials. In addition, we found a higher prevalence of UFM calls in adult–juvenile trials compared with adult–adult trials. Because we did not always know the identity of the caller, we cannot say whether these results represent juveniles emitting more social calls, adults producing more social calls in the presence of juveniles, or both. One possible explanation is that juvenile-adult dyads create a different social dynamic than adult pairs, perhaps resulting in increased likelihood of appeasement-related calling by juveniles.

There is a paucity of literature reporting social calls from *E. fuscus*, but papers on vocal development in pups, and including some calls from adults, describe vocalizations resembling CS, LFM, and QCF calls (Moss, 1988) or U and LFM calls (Monroy et al., 2011). Some of the calls we recorded also show similarities to those Gadziola et al. (2012) recorded from crawling/roosting adult and juvenile bats. For example, their DFMs syllable, which was recorded in an aggression context, is structurally similar to our SFM, except that the former were usually emitted as a multi-syllabic call. Low frequency, multi-harmonic, calls resembling those described as aggressive calls by Gadziola et al. (2012; e.g., rBNBs and rBNBl) were not emitted by flying *E. fuscus* in our study but were often emitted when bats were being handled by humans. The time-frequency characteristics of these calls are distinct from short duration (0.5–1 ms) buzz-like calls, which we excluded on the basis that social buzzes may not be easily distinguished from feeding, inspection, or landing buzzes. Gadziola et al. (2012) recorded calls very similar to our LFM calls, including couplets of calls (DFMl, shalDFMl, DFMl-QCFl, and DFMl-QCF-UFM), in an appeasement context. Additional call types were similar in some attributes (e.g., call shape) but not in others (e.g., call frequency) to the calls described in this paper. In general, the calls Gadziola et al. (2012) recorded from crawling/roosting bats were lower in frequency than the vocalizations we recorded from flying bats of the same species. Bats in flight may be more likely to employ social calls with frequencies overlapping with those of their echolocation pulses so as to use the echo return information from social vocalizations. Another possible explanation for use of higher frequency social calls in flight is that flying bats might reflexively increase the tension on their vocal membranes as they would to produce sonar calls. That some calls were recorded exclusively in a flying or a crawling/roosting context highlights the breadth of potential information bats could convey via communicative vocalizations and provides further evidence of context-specific use of such calls.

While relatively few papers present social calls from flying, foraging bats, each of the call types described here shares some spectral attributes with communicative calls recorded from other bat species in various contexts. For example, *Desmodus rotundus* isolation calls and calls emitted by mothers searching for their young (Fenton, 1985), as well as the alarm calls of *Tadarida brasiliensis* (Bohn et al., 2008), each contain portions that rise in frequency, as does our UFM. Chevron-shaped (CS) calls are produced by juvenile *Pteropus poliocephalus* (Nelson, 1964) in an isolation and location context, as well as by *Saccopteryx bilineata* in their territorial song (Behr and von Helversen, 2004) and by *T. brasiliensis* in directive and face rub calls (Bohn et al., 2008). Double-note calls emitted by *Myotis lucifugus* in maternity colonies and during swarming contain a portion resembling our U call (Barclay et al., 1979). As noted, our LFM resembles isolation calls, including those of *M. lucifigus* (Barclay et al., 1979), as well as showing similarity to a marking call of *T. brasiliensis* (Bohn et al., 2008), and social calls emitted by *M. bechsteinii* in maternity roosts and in flight (Pfalzer and Kusch, 2003) and *Pteronotus parnelli* (Kanwal et al., 1994). Our SFM and QCF calls bear some resemblance to the irritation and mounting calls, respectively, of *T. brasiliensis* (Bohn et al., 2008), and *P. parnelli* also produce lower frequency QCF calls in a social context (Kanwal et al., 1994). Finally, our FMB is similar in structure to individually-specific contact calls emitted by *Antrozous pallidus* (Arnold and Wilkinson, 2011). It should be noted that while the calls we describe here share some structural similarities with calls emitted by other species, the frequency ranges may not overlap. The variety of call types emitted, with calls of similar shape being used in very different contexts by different species, indicates that caution must be used when attempting to generalize call function based on spectral features alone.

This study uncovered a rich repertoire of social calls produced by flying big brown bats, *Eptesicus fuscus*, one of the most studied bats in North America. We found that males produced more social calls and that bats flew in closer proximity when emitting UFM, SFM, LFM, and QCF calls. By varying the context in which pairs of bats flew, we were able to determine that some call types are produced in a foraging-related context. These findings highlight the importance of inter-individual acoustic communication in bats as they forage, and lay the foundation for future research on the functional role of bat social calls in a variety of settings, both in the lab and the field.

### Conflict of interest statement

The authors declare that the research was conducted in the absence of any commercial or financial relationships that could be construed as a potential conflict of interest.
